# An Estimate of the Burden of Fungal Disease in Norway

**DOI:** 10.3390/jof4010029

**Published:** 2018-02-21

**Authors:** Ingvild Nordøy, Liv Hesstvedt, Cecilie Torp Andersen, Haima Mylvaganam, Nicola I. Kols, Birgit M. Falch, Ståle Tofteland, Fredrik Müller, David W. Denning

**Affiliations:** 1 Section for Clinical Immunology and Infectious Diseases, Oslo University Hospital, Rikshospitalet, 0372 Oslo, Norway; 2 Research Institute for Internal Medicine, Oslo University Hospital, Rikshospitalet, 0372 Oslo, Norway; uxzhcl@ous-hf.no; 3 Department of Microbiology, Oslo University Hospital, 0372 Oslo, Norway; ceanders@ous-hf.no (C.T.A.); Fredrik.müller@ous-hf.no (F.M.); 4 Department of Microbiology, Haukeland University Hospital, Bergen, 5021 Bergen, Norway; haima.mylvaganam@helse-bergen.no; 5 Department of Microbiology, St. Olav’s Hospital, 7030 Trondheim, Norway; nicola.isabelle.kols@stolav.no; 6 Department of Microbiology, University Hospital of Northern Norway, 9019 Tromsø, Norway; Birgit.margrethe.falch@unn.no; 7 Department of Microbiology, Sørlandet Hospital, 4615 Kristiansand, Norway; staale.tofteland@sshf.no; 8 Department of Microbiology, Oslo University, 0371 Oslo, Norway; 9 National Aspergillosis Centre, Wythenshawe Hospital and The University of Manchester, Manchester M13 9PL, UK; ddenning@manchester.ac.uk; 10 Leading International Fungal Education (LIFE), Cheshire SK10 9AR, UK

**Keywords:** epidemiology, fungal burden, allergic bronchopulmonary aspergillosis (ABPA), severe asthma with fungal sensitization (SAFS), *Pneumocystis*, *Candida*, aspergillosis, mucorales, Norway

## Abstract

The aim of this study was to examine the burden of fungal disease in Norway, contributing to a worldwide effort to improve awareness of the needs for better diagnosis and treatment of such infections. We used national registers and actual data from the Departments of Microbiology from 2015 and estimated the incidence and/or prevalence of superficial, allergic and invasive fungal disease using published reports on specific populations at risk. One in 6 Norwegians suffered from fungal disease: Superficial skin infections (14.3%: 745,600) and recurrent vulvovaginal candidiasis in fertile women (6%: 43,123) were estimated to be the most frequent infections. Allergic fungal lung disease was estimated in 17,755 patients (341/100,000). *Pneumocystis jirovecii* was diagnosed in 262 patients (5/100,000), invasive candidiasis in 400 patients (7.7/100,000), invasive aspergillosis in 278 patients (5.3/100,000) and mucormycosis in 7 patients (0.1/100,000). Particular fungal infections from certain geographic areas were not observed. Overall, 1.79% of the population was estimated to be affected by serious fungal infections in Norway in 2015. Even though estimates for invasive infections are small, the gravity of such infections combined with expected demographic changes in the future emphasizes the need for better epidemiological data.

## 1. Introduction

Fungal diseases are common with variable morbidity ranging from a toenail infection causing cosmetic worry to invasive disease with a relatively high mortality rate among infectious diseases. The incidence of invasive fungal infections is highly dependent on patients with immunocompromised states. Therefore, societies where acquired immune deficiency syndrome (AIDS) and tuberculosis are of major concern are in dire need of rapid and accurate diagnosis and availability of antifungal drugs to acceptable prices.

In the western world, many report an increased incidence of invasive fungal infections, but apart from candidemias, few epidemiological studies exist on other fungal infections [1,2]. Due to improvement in the general health and medical care in this part of the world, we expect more patients and in particular an increasing proportion of the elderly to live their life longer in an immunocompromised state. Thus, the predicament is that fungal infections will become a bigger problem in years ahead.

Estimates of the fungal burden in various countries have been made in order to increase awareness of these diseases in the medical and political community. Due to lack of incidence and prevalence data on fungal infections, estimates have been made based on extrapolation of data from known conditions rendering the patient susceptible to these infections combined with actual data from publications in smaller populations. In Norway, national surveillance of candidemia has been ongoing since 1991 [3,4,5]. No reports on the epidemiology of other fungal disease exist. In this study, we aim to estimate the burden of fungal disease based on actual and estimated data from 2015.

## 2. Materials and Methods

Data on specific populations were obtained from national data registers concerning (1) demography [6]; (2) chronic obstructive pulmonary disease (COPD) and tuberculosis [7]; (3) solid organ transplantation (SOT) [8], hematopoietic stem cell transplantation (HSCT) (personal communication from Tobias Gedde-Dahl, Department of Hematology, Oslo University Hospital, Oslo, Norway); (4) hematological and lung cancers [9]; (5) cystic fibrosis (CF) [10]; (6) HIV [11] and (7) candidemia [12]. Prevalence of fungal disease was calculated based on previously published epidemiological studies on superficial skin infection, vulvovaginal candidiasis, fungal keratitis, chronic pulmonary aspergillosis, allergic bronchopulmonary aspergillosis, severe asthma with fungal sensitization and invasive aspergillosis [13,14,15,16,17,18,19,20,21]. Recurrent vulvovaginal candidiasis (RVVC) was defined at 4 or more episodes annually [14]. Data on candidemia was retrieved from the Norwegian Mycological Reference Laboratory at Oslo University Hospital, as all *Candida* isolates found in blood cultures are collected here. Data on infections due to *Pneumocystis jirovecii, Cryptococcus* ssp., mucorales and sporadic fungal infections (*Histoplasma capsulatum*, *Blastomyces dermatitidis*, *Coccidioides* ssp., *Paracoccidioides brasiliensis*, and *Talaromyces marneffei*) were collected from the 6 medical microbiological laboratories in Norway where these infections are diagnosed. They reported their findings on these specific agents in 2015 answering a form sent to each one and using filtering systems where the analyses (culture, PCR, antigen-test and immunofluorescence) and agents (*P. jirovecii*, Mucorales ssp., *Cryptococcus* ssp., *H. capsulatum*, *B. dermatitidis*, *Coccidioides* ssp., *P. brasiliensis*, and *T. marneffei*) were retracted.

## 3. Results

### 3.1. Country Profile

Norway had a population of 5,213,985 as of January 1st 2016 (2,625,111 men), with 21.6% being under 18 years of age and 10.1% being 70 years of age or older. The number of women between 15 and 50 years of age was 1,235,379 (23.6% of the population). The life expectancy of men and women was 80.4 and 84.2 years, respectively. The standard of living was high with a gross national product per capita in 2015 of $74,482 [22]. Education and health care are provided almost free of charge to all inhabitants, as they are covered through taxes.

### 3.2. Superficial Fungal Infections

No data on incidence/prevalence of superficial fungal infections including oral candidiasis, *Candida* oesophagitis, RVVC and keratitis, has been reported from Norway. Based on previous worldwide estimates, a 14.3% incidence of superficial skin disease would result in 745,600 infections/year (Table 1) [13].

Based on unpublished data from the largest HIV-clinic in Norway, 9% of the patients were not receiving anti-retroviral therapy (personal communication from Bente Magny Bergersen, Department of Internal Medicine, Oslo University Hospital, Oslo, Norway). In 2015, 3,435 patients with HIV infection lived in Norway. Assuming 20% of untreated patients and 0.5% of treated patients had esophageal candidiasis (*N* = 76), and 90% of untreated patients had oral *Candida* infection (*N* = 278), 354 HIV patients had *Candida* in the upper GI-tract (Table 1) [23,24].

Using an estimate of 6% prevalence of RVVC in women aged 15 to 50 years, 74,123 would have RVVC in 2015 (Table 1) [14]. Based on Danish data from Nielsen et al., a minimum of 0.6 cases per million of fungal keratitis was estimated, leading to an estimate of 3 cases of fungal keratitis in 2015 (Table 1) [15].

### 3.3. Respiratory Disease

The prevalence of asthma in the Norwegian population is unknown, but applying the Danish prevalence of 6.4% of the adult population (*N* = 261,541) and an estimated 2.5% of these with allergic bronchopulmonary aspergillosis (ABPA), we had 6,539 asthmatic patients in Norway with ABPA in 2015 (Table 1) [16,25]. In addition, 17.7% of adult patients and 8.2% of children with cystic fibrosis (CF) were estimated to have ABPA [18]. In 2015, we had 195 adult and 130 children living with CF, adding 46 patients with ABPA, rendering a total of 6,585 patients (Table 1).

Severe asthma with fungal sensitization (SAFS) is a new and neglected disease. We calculated estimates based on the number of patients with severe asthma (10% of the total). Estimates have differed, but in a study by O’Driscoll et al., 43% of patients with severe asthma having SAFS may be a modest estimate in patients with severe asthma, giving a number of 11,246 patients (Table 1) [21,26]. SAFS is rare in children. *Aspergillus* bronchitis was estimated in 30% of adult CF patients, rendering an estimate of 59 patients [18]. *Aspergillus* bronchitis also occurs in other patients, notably those with bronchiectasis, but a burden estimate is not possible currently.

Chronic pulmonary aspergillosis (CPA) is usually associated with underlying pulmonary disease. Globally, tuberculosis is the main underlying cause, but this is a rare infection in Norway today. The Norwegian Institute of Public Health reported 318 patients with tuberculosis in 2015. Of these, 224 (70%) had pulmonary tuberculosis with a <1% mortality rate. Twenty-seven (12%) were estimated to have cavitating disease, while 195 did not [17]. Based on data from Denning et al., 22% of patients with cavitating disease develop CPA and 2% of those without, resulting in 8 patients with CPA [17]. Approximately 15% will die annually from their CPA, resulting in a 5-year prevalence of 35. As pulmonary tuberculosis accounts for 16% of CPA cases, this gives a total estimate of prevalence of 219 with CPA (Table 1) [19].

*Pneumocystis* pneumonia (PCP) was previously associated with HIV infection. In 2015, 221 patients were diagnosed with HIV in Norway, 11 with AIDS. The total living HIV population amounted to 3435 patients. An estimate of 4 PCPs among HIV patients was made, based on Danish data from Thorstensson et al., where 37% of patients with AIDS-defining illness presented with PCP [27]. However, a total of 262 positive *P. jirovecii* PCRs from 262 patients were registered overall in 2015, implying non-HIV patients to be more at risk for PCP (Table 1). Data on the correlation to PCP in these patients was not obtained.

### 3.4. Invasive Candidiasis and Aspergillosis

Two hundred candidemias were registered in 2015 [12] (Table 1). Assuming the number of diagnosed candidemias reflects only 50% of the invasive *Candida* infections, we report a total number of 400 invasive *Candida* infections, which includes 43 *Candida* infections estimated in patients with hematological malignancies (Table 1) [28]. Invasive *Aspergillus* (IA) infection was estimated in transplant recipients, in patients with hematological malignancies and lung cancer based on estimates according to others (Table 2) [29,30,31,32].

In 519 transplant recipients, 17 patients were estimated to have IA (Table 2). In 2845 patients with hematological malignancies and in 3125 with lung cancer, 83 and 81 patients, were estimated to have IA, respectively (Table 2). Based on data from others, approximately 1.3% of admitted patients with severe COPD also developed IA (Table 2) [20,33]. In 2015, 7472 patients were admitted due to COPD, giving an estimated number of 97 IA patients, resulting in a total of 278 patients with IA.

### 3.5. Other Fungal Infections

In 2015, mucorales were reported in 7 patients, while *Cryptococcus* spp. or fungal infections particular to other geographic regions were not observed (<0.1/100,000).

## 4. Discussion

In this study, we found that the estimated overall burden of fungal disease in Norway is high, affecting almost 16 percent of the population. However, excluding superficial skin infections, only 1.79% of the population is affected based on actual numbers from the Microbiological laboratories and estimates. Invasive infections were not associated with HIV or tuberculosis, but were associated with other immunocompromising conditions such as cancer, autoimmune diseases or following organ transplantation.

We do not have data on superficial skin infections and therefore present estimates. We have used estimates by Vos et al., who predicted a 14.3% prevalence [13]. However, Sigurgeirsson and Baran report in a review considerably lower numbers of onychomycosis both from population-based and hospital-based studies from Europe and North America, 4.3% and 8.9%, respectively [34]. In Norway, nail-infections are expected to be the most common superficial infection. The numbers we present may therefore be an overestimation of the problem of fungal superficial infections.

Respiratory infections are also a result of estimates, except for data on PCP. Exact numbers on the prevalence of asthma in the adult population are unknown, which is why we used Danish data believed to be similar as the basis for both ABPA and SAFS. CPA is more difficult to assess, as estimates are based on the prevalence of pulmonary tuberculosis, which is rare. CPA may therefore be underestimated, as COPD and other autoimmune conditions such as sarcoidosis and others may be more relevant conditions to consider.

Regarding PCP, this is based on data from the 6 Microbiological laboratories which performed *P. jirovecii* PCR in Norway in 2015. Mortensen et al. estimates only 1.5/100,000 PCPs from Denmark in 2013 [35]. From Germany and UK the numbers are even lower, 1.2/100,000 and 0.33–0.93/100,000 respectively [36,37]. From France a national retrospective study using patient records reported an incidence of 1.5/100,000 [38]. We report 262 PCR-positive cases giving an incidence of 5/100,000. Carriage among healthy individuals has been reported [39]. It has also been reported from patients not suspected of having PCP, though a recent study suggests that colonization in patients may in fact represent a mild disease [40,41]. The PCR method is extremely sensitive. Some of our findings certainly may represent colonization by the fungus and not clinical infection. However, having been unable to examine clinical data, we cannot conclude on this matter. While our PCP numbers are probably too high, we believe other reported numbers may be too low. Based on French data, 41% of PCP was associated with HIV infection, while 59% had other immunosuppressive diseases [42]. In Norway only 4 of the 262 *P. jirovecii* PCR-positive samples were estimated to be from AIDS patients. Even if only 50% of the total PCR–positive numbers indicated clinical infection, the patients at risk in Norway, and probably in other western countries, are many. Further studies in this field are needed.

In Norway, candidemia has been followed closely since 1991. The incidence is low (3–4/100,000) and has been stable for the last years [4]. Assuming that the laboratory-verified candidemia represent only 50% of the actual invasive Candida infections, we have doubled the number to make a more accurate estimate of the numbers [28]. Why the incidence in Norway has stayed low is uncertain. We do see an extensive and increasing use of antifungal drugs, but the use of prophylaxis is reserved for extreme premature or patients with leukemia or undergoing HSCT or pancreas transplantation [43]. However, the use of broad-spectrum antibiotics and antibiotics in general is probably more conservative compared to other countries, due to lower levels of antibiotic resistance. Infection control procedures are strict and generally well observed. These factors may indeed prevent the establishment of fungal infections. However, with the demographic shift with an increasing elderly population, and knowing this patient group represents over 50% of patients with candidemia [1,3,4], we expect these infections to be on the rise.

Following invasive candidiasis and probably PCP, invasive aspergillosis is believed to be one of the more “common” invasive fungal diseases in Norway. However, here we lack specific data. This is partly due to the difficulty in diagnosing this infection, as *Aspergillus* sp. enters the human body through the airways, and causes a relatively silent infection. Positive tests may indicate spores that are being inhaled without implying infection. Whether our estimates are too low or high is difficult to say, but these infections, though few, are often fatal and expensive, and may be on the rise given the demographic outlook and more aggressive use of immune-modulating drugs. In 2015 we had 7 cases of invasive mucormycosis. This is a high number, but the incidence is suspected to be on the rise, probably due to better diagnostics. Though Norwegians are travelling people, there are rarely reports of particular infections from geographic areas.

This study is part of a global study initiated by Leading International Fungal Education [44], which has launched the initiative to calculate the fungal burden in countries in order to ascertain the public health importance of fungal infections and the need to improve diagnostics and treatment. Estimates have demonstrated that worldwide deaths due to fungal infections (>1,350,000) are as high as those of tuberculosis (1,400,000) and significantly higher than malaria (445,000) [45,46]. The drawbacks of the study are several. Diagnostic limitations on reported data are still relevant. Regarding invasive infections such as candidemia, we can assume both an underdiagnosis and overdiagnosis to occur. Furthermore, much of the data are estimates, again based on estimates from other countries, and may not be a precise tally of the actual numbers of these infections in Norway, where the climate is cold, wealth and educational levels are high and the expected age of living exceeds 80 years for both sexes. However, even given a low incidence of invasive infections, the morbidity, mortality and economic costs associated with them warrants better epidemiological data.

## Figures and Tables

**Table 1:** The burden of fungal disease in Norway in 2015.

Disease Due to Fungal Infection	Number of Infections per Underlying Disorders							Total Burden	Rate/100,000
	No Underlying Disorder	HIV/AIDS		Respiratory Disease			Cancer, Transplantation, Others *		
**Superficial infection**									
Skin/nail/hair infection	745,600		n.a		n.a.	n.a		745,600	14,338/100,000
Oral *Candida* infection	n.a.		278		n.a.	n.a.		278	5.3/100,000
Esophageal candidosis	n.a.		76		n.a.	n.a.		76	1.5/100,000
Recurrent vaginitis	74,123		n.a.		n.a.	n.a.		74,123	2,863/100,000 woman
**Allergic disease**									
Allergic bronchopulmonary aspergillosis	n.a.		n.a.		6585	n.a.		6585	127/100,000
Severe asthma with fungal sensitization	n.a.		n.a.		11,246	n.a		11,246	332/100,000
**Invasive/systemic/deep fungal infection**									
Candidemia	n.a.		n.a.		n.a.	n.a.		200	3.8/100,000
Other invasive *Candida* infections	n.a.		n.a.		n.a.	n.a.		200	3.8/100,000
Other invasive yeast infections	n.a.		n.a.		n.a.	n.a.		5	<0.1/100,000
Cryptococcal meningitis	n.a.		0		n.a.	0		0	<0.1/100,000
*Pneumocystis jirovecii* pneumonia	n.a.		4		258	n.a.		262	5/100,000
Invasive aspergillosis	n.a.		n.a.		97	181		278	5.3/100,000
Chronic pulmonary aspergillosis	n.a.		n.a.		219	n.a.		219	4/100,000
Mucormycosis	n.a.		n.a.		n.a.	7		7	0.1/100,000
Other mould infections	n.a.		n.a.		n.a.	n.a.		8	0.1/100,000
**Total**	**819,723**		**358**		**18,405**	**188**		**839,087**	

* Others: other immunodeficiencies; n.a.: not available.

**Table 2:** Estimate of invasive aspergillosis in Norway 2015.

Predisposing Conditions	Number of Cases	IA * Occurrence %	Number of IA
**Organ transplantations**			
Allogeneic HSCT **	72	10	7
Renal	254	1	3
Liver	86	4	3
Heart	37	6	2
Lung	34	6	2
Pancreas	31		0
Pancreatic Islet cells	5		0
**Hematological malignancies**	2845	2.9	83
**Lung cancer**	3125	2.6	81
**Severe chronic obstructive pulmonary disease**	7472 admitted	1.3	97
**Total**	13,961		278

* IA: Invasive Aspergillosis; ** HSCT: Hematopoietic Stem Cell Transplantation.

## References

[1] Arendrup M.C. (2010). The epidemiology of invasive candidiasis. Curr. Opin. Crit. Care.

[2] Lortholary O., Gangneux J.P., Sitbon K., Lebeau B., de Monbrison F., Le Strat Y., Coignard B., Dromer F., Bretagne S. (2011). The French Mycosis Study Group. Epidemiological trends in invasive aspergillosis in France: the SAIF network (2005–2007). Clin. Microbiol. Infect..

[3] Sandven P., Bevanger L., Digranes A., Haukland H.H., Mannsåker T., Gaustad P. (2006). Norwegian Yeast Study Group. Candidemia in Norway (1991 to 2003): Results from a nationwide study. J. Clin. Microbiol..

[4] Hesstvedt L., Gaustad P., Andersen C.T., Haarr E., Hannula R., Haukland H.H., Hermansen N.O., Larssen K.W., Mylvaganam H., Ranheim T.E. (2015). The Norwegian Yeast Study Group. Twenty-two years of candidaemia surveillance: Results from a Norwegian national study. Clin. Microbiol. Infect..

[5] Berdal J-E., Haagensen R., Ranheim T., Bjørnholt J.V. (2014). Nosocomial candidemia; risk factors and prognosis revisited; 11 years experience from a Norwegian secondary hospital. Plos ONE.

[6] Statistisk sentralbyrå. https://www.ssb.no/en/.

[7] Folkehelseinstituttet. https://www.fhi.no/.

[8] Scandiatransplant. http://www.scandiatransplant.org/.

[9] KREFT registered. https://www.kreftregisteret.no/.

[10] Norwegian Centre for Cystic Fibrosis. www.oslo-universitetssykehus.no.

[11] Nasjonalt servicemiljø for medisinske kvalitetsregistre. https://www.kvalitetsregistre.no/.

[12] (2016). NORM/NORM-VET 2015.

[13] Vos T., Flaxman A.D., Naghavi M., Lozano R., Michaud C., Ezzati M., Shibyya K., Salomon J.A., Abdalla S., Aboyans V. (2012). Years lived with disability (YLDs) for 1160 sequelae of 289 diseases and injuries 1990–2010: A systematic analysis for the Global Burden of Diseases Study 2010. Lancet.

[14] Foxman B., Muraglia R., Dietz J.P., Sobel J.D., Wagner J. (2013). Prevalence of Recurrent Vulvovaginal Candidiasis in 5 European Countries and the United States: Results from an internet panel survey. J. Low. Genit. Tract. Dis..

[15] Nielsen S.E., Nielsen E., Julian H.O., Lindegaard J., Højgaard K., Ivarsen A., Hjortdal J., Heegaard S. (2015). Incidence and clinical characteristics of fungal keratitis in a Danish population. Acta. Ophtalmol..

[16] Denning D.W., Peuvry A., Cole D.C. (2013). Global burden of allergic bronchopulmonary aspergillosis with asthma and its complication chronic pulmonary aspergillosis in adults. Med. Mycol..

[17] Denning D.W., Peuvry A., Cole D.C. (2011). Global burden of chronic pulmonary aspergillosis as a sequel to pulmonary tuberculosis. Bull. World Health Organ..

[18] Armstead J., Morrisi J., Denning D.W. (2014). Multi-country estimate of different manifestations of aspergillosis in Cystic Fibrosis. Plos ONE.

[19] Denning D.W., O’Driscoll B.R., Hogaboam C.M., Bowyer P., Niven R.M. (2006). The link between fungi and severe asthma: A summary of the evidence. Eur. Respir. J..

[20] Lopez-Campos B.J.L., Fernandez G.J., Lara B.A., Perea-Milla L.E., Moreno L., Cebrián G.J.J., Garcia J.J.M. (2002). Analysis of admissions for chronic obstructive pulmonary disease in Andalusia 2000. Arch. Bronchopneumol..

[21] Brown G.D., Denning D.W., Gow N.A.R., Levitz S.M., Netea M.G., White T.C. (2012). Hidden Killers: Human Fungal Infections. Sci. Transl. Med..

[22] The World Bank. www.worldbank.org.

[23] Smith E., Orholm M. (1990). Trends and patterns of opportunistic diseases in Danish AIDS patients 1980–1990. Scand. J. Infect. Dis..

[24] Matee M.I., Scheutz F., Moshy J. (2000). Occurrence of oral lesions in relation to clinical and immunological status among HIV-infected adult Tanzanians. Oral Dis..

[25] Von Bülow A., Kriegbaum M., Backer V., Porsbjerg C. (2014). Severe asthma—Where are we today?. Ugeskrift Laeger.

[26] O’Driscoll B.R., Powell G., Chew F., Niven RM., Miles J.F., Vyas A., Denning D.W. (2009). Comparison of skin prick tests with specific serum immunoglobulin E in the diagnosis of fungal sensitization in patients with severe asthma. Clin. Exp. Allergy.

[27] Thorstensson K., Ladelund S., Jensen-Fangel S., Larsen M.V., Johansen I.S., Kazenstam T.L., Pedersen G., Storgaard M., Obel N., Lebech A.M. (2012). Impact of gender on the risk of AIDS-defining illnesses and mortality in Danish HIV-1-infected patients: A nationwide cohort study. Scand J. Infect. Dis..

[28] Clancy C.J., Nguyen M.H. (2013). Finding the Missing 50% of invasive candidiasis: How nonculture diagnostics will improve understanding of disease spectrum and transform patients care. Clin. Infect. Dis..

[29] Herbrecht R., Bories P., Moulin J-C., Ledoux M-P., Letscher-Bru V. (2012). Risk stratification for invasive aspergillosis in immunocompromised patients. Ann. N. Y. Acad. Sci..

[30] Iversen M., Burton C.M., Vand S., Skovfoged L., Carlsen J., Milman N., Andersen C.B., Rasmussen M., Tvede M. (2007). Aspergillus infection in lung transplant patients: Incidence and prognosis. Eur. J. Clin. Microbiol. Infect. Dis..

[31] Pagano L., Caira M., Candoni A., Offidani M., Fianchi L., Martino B., Pastore D., Picardi M., Bonini A., Chierchini A. (2006). The epidemiology of fungal infections in patients with haematological malignancies. Haematologica.

[32] Yan X., Li M., Jiang M., Zou L-Q., Luo F., Jiang Y. (2009). Clinical characteristics of 45 patients with invasive pulmonary aspergillosis. A retrospective analysis of 1711 lung cancer cases. Cancer.

[33] Guinea J., Torres-Narbona M., Gijón P., Munoz P., Pozo F., Peáez T., de Miguel J., Bouza E. (2010). Pulmonary aspergillosis in patients with chronic obstructive pulmonary disease: Incidence, risk factors, and outcomes. Clin. Microbiol. Infect..

[34] Sigurgeirsson B., Baran R. (2014). The prevalence of onychomycosis in the global population: A literature study. J. Eur. Acad. Dermatol. Venereal..

[35] Mortensen K.L., Denning D.W., Arendrup M.C. (2015). The burden of fungal disease in Denmark. Mycoses.

[36] Ruhnke M., Groll A., Mayser P., Schaller M., Mendling W., Ullmann A., Denning D.W. (2015). The University of Manchester in association with the LIFE program. Estimated burden of fungal infections in Germany. Mycoses.

[37] Pegorie M., Denning D.W., Welfare W. (2017). Estimating the burden of invasive and serious fungal disease in the United Kingdom. J. Infect..

[38] Bitar D., Lortholary O., Le Strat Y., Nicolau J., Coignard B., Tattevin P., Che D., Dromer F. (2014). Population-based analysis of invasive fungal infections, France, 2001–2010. Emerg. Infect. Dis..

[39] Ponce C.A., Gallo M., Bustamante R., Vargas S.L. (2010). *Pneumocystis* colonization is highly prevalent in the autopsied lungs of the general population. Clin. Infect. Dis..

[40] Peters S.E., Wakefield A.E., Banerji S., Hopkin J.M. (1992). Quantification of the detection of *Pneumocystis carinii* by DNA amplification. Mol. Cell Probes.

[41] Prickartz A., Lüsebrink J., Khalfaoui S., Schildgen O., Schildgen V., Windisch W., Brockmann M. (2016). Low Titer *Pneumocystis jirovecii* Infections: More than Just Colonization?. J. Fungi.

[42] Roux A., Canet E., Valade S., Gangneux-Robert F., Hamane S., Lafabrie A., Maubon D., Debourgogne A., Le Gal S., Dalle F. (2014). *Pneumocystis jirovecii* pneumonia in patients with or without AIDS, France. Emerg. Infect. Dis..

[43] Hesstvedt L., Arendrup M.C., Poikonen E., Klingspor L., Friman V., Nordøy I. (2017). Differences in the epidemiology of candidemia in the Nordic countries—What is to blame?. Mycoses.

[44] Leading International Fungal Education. http://www.life-worldwide.org/..

[45] (2017). Global Tuberculosis Report 2017.

[46] (2017). World Malaria Report 2017.

